# Supplementary material for: Loss of function of chromatin remodeler OsCLSY4 leads to RdDM-mediated mis-expression of endosperm-specific genes affecting grain qualities
Source: PLoS Genet. 2025 Dec 1;21(12):e1011956. doi: 10.1371/journal.pgen.1011956 (PMC12680349; doi:10.1371/journal.pgen.1011956)
Supplement: S1 Fig — (PDF) [file pgen.1011956.s001.pdf]

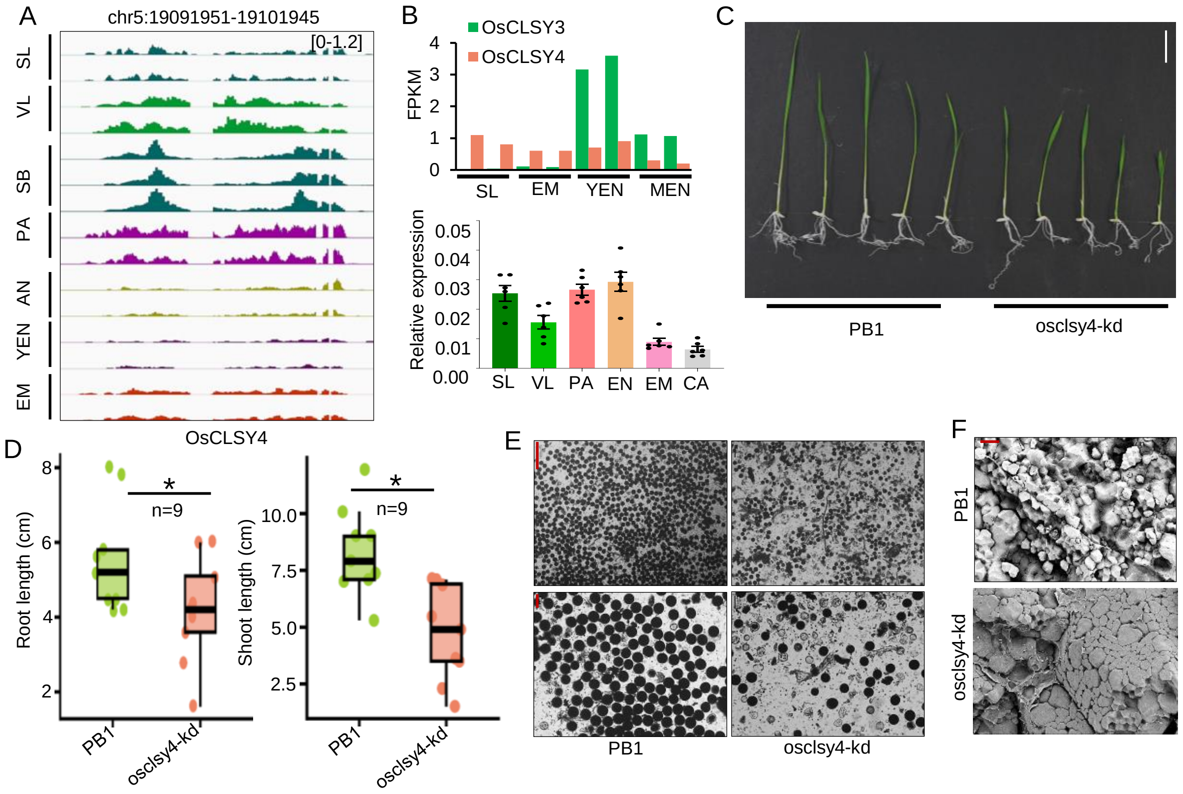

S1_Fig: Phenotypes of osclsy4-kd plants.
(A) IGV screenshot representing expression of OSCLSY4 gene across different rice tissues. SL-seedling (GSE130168), VL- vegetative leaf (GSE138705), SB-shootbase (GSE131319), PA-panicle (GSE180457), AN-anther (GSE180457), YEN-young endosperm (GSE229959), EM-embryo (GSE229959). (B) Barplots showing expression of OsCLSY4 and OsCLSY3 across different rice tissues. MEN-Mature endosperm (GSE229959) in transcriptomes (top). RTqPCR analysis showing expression of OsCLSY4 in different tissues, CA-calli. *OsGAPDH* served internal control. (C) Image showing phenotypes of PB1 and osclsy4-kd 10 days old seedlings. Scale bar (SB)-2 cm. (D) Boxplots showing root and shoot length of PB1 and osclsy4-kd seedlings. (E) Pollen viability assay in PB1 and osclsy4-kd. SB-100 µm. (F) Electron microscopy images of PB1 and osclsy4-kd EN. SB-10 µm.
