# Supplementary material for: Loss of function of chromatin remodeler OsCLSY4 leads to RdDM-mediated mis-expression of endosperm-specific genes affecting grain qualities
Source: PLoS Genet. 2025 Dec 1;21(12):e1011956. doi: 10.1371/journal.pgen.1011956 (PMC12680349; doi:10.1371/journal.pgen.1011956)
Supplement: S2 Fig — (PDF) [file pgen.1011956.s002.pdf]

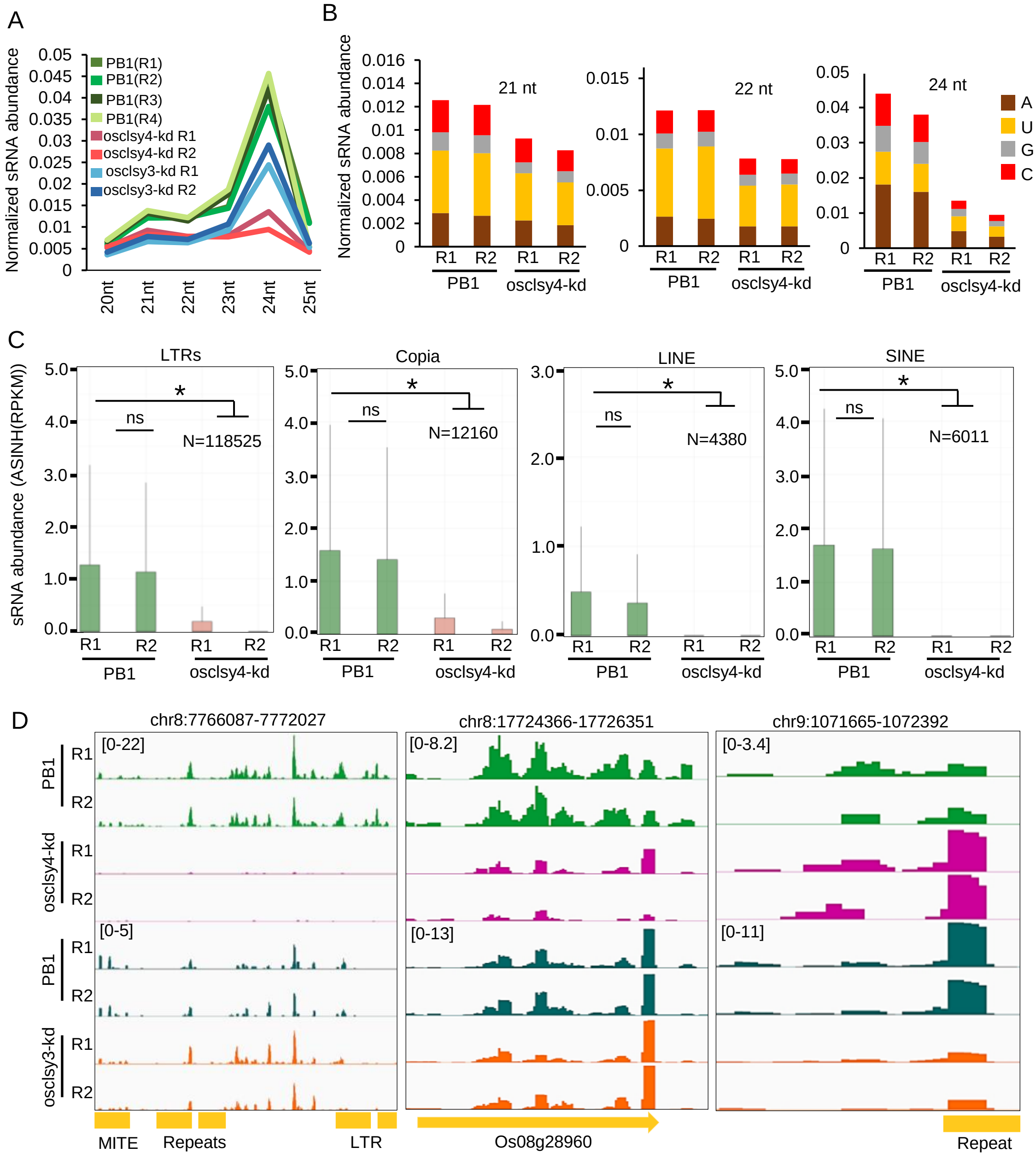

S2 _Fig: Endosperm-sRNAs are regulated by both OsCLSYs.
(A) Plot showing expression of 21-25 nt sRNAs in PB1 and individual kd lines in EN. (B) Stacked barplot showing abundance of first 5' nucleotide of mapped sRNAs in WT and clsy4-kd EN. (C) Boxplots representing expression of 23-24 nt sRNAs across different TEs. *-significant. ns-non-significant (Wilcoxon test $p < 0.01$). (D) IGV screenshots showing expression of 23-24 nt sRNAs in different CLSY-dependent sRNA loci.
