# Supplementary material for: Loss of function of chromatin remodeler OsCLSY4 leads to RdDM-mediated mis-expression of endosperm-specific genes affecting grain qualities
Source: PLoS Genet. 2025 Dec 1;21(12):e1011956. doi: 10.1371/journal.pgen.1011956 (PMC12680349; doi:10.1371/journal.pgen.1011956)
Supplement: S3 Fig — (PDF) [file pgen.1011956.s003.pdf]

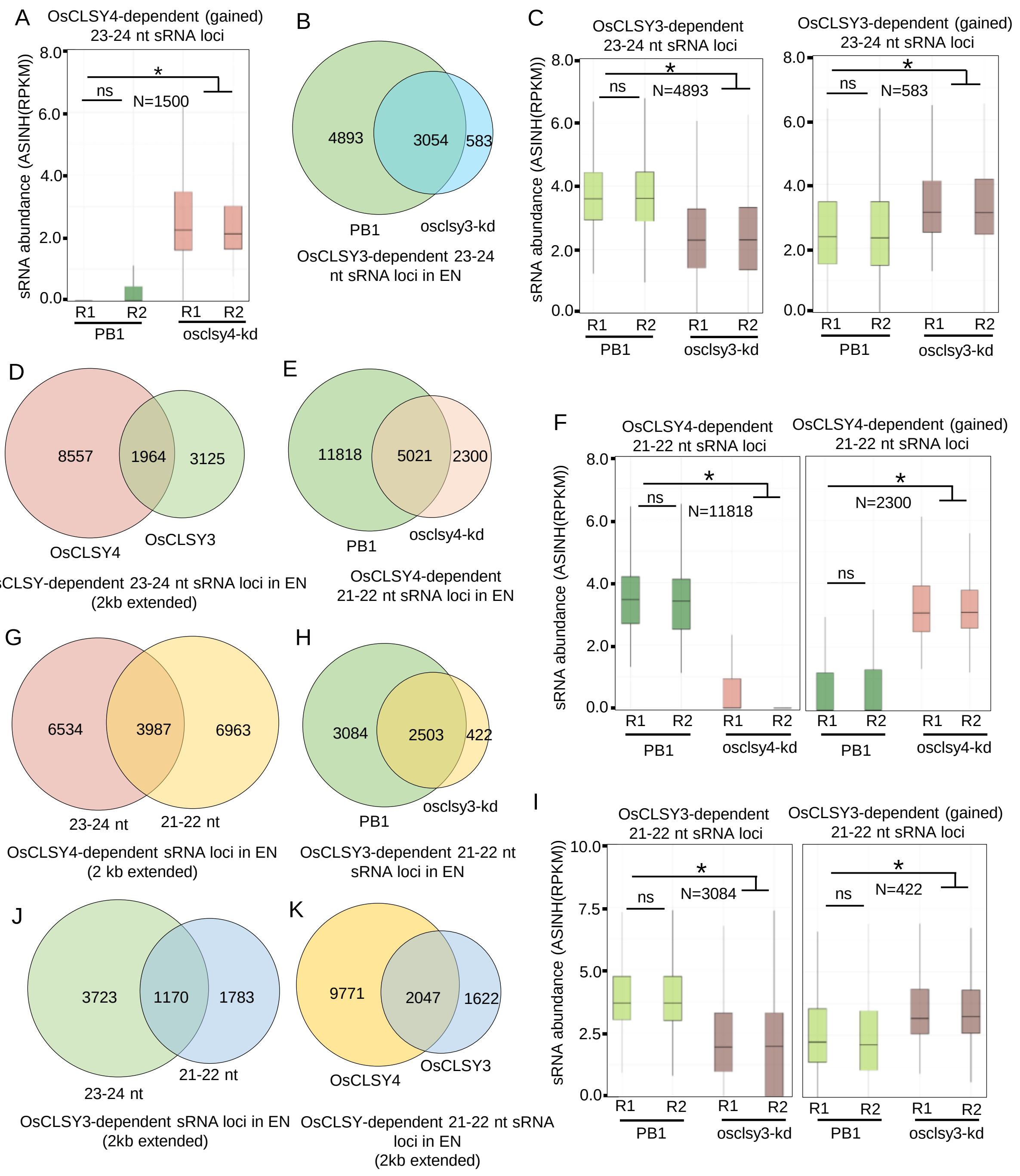

S3 _Fig: OsCLSYs regulate expression of 21-22 nt sRNAs in endosperm.
(A) Boxplots representing OsCLSY4 dependent 23-24 nt gained sRNA loci in EN. (B) Venn diagram showing OsCLSY3 dependent 23-24 nt sRNA loci in EN. (C) Boxplots representing abundance of 23-24 nt sRNAs from OsCLSY3 dependent 23-24 nt sRNA loci (lost and gained, respectively). (D) Venn diagram showing overlap between OsCLSY4 and OsCLSY3 dependent 23-24 nt sRNA loci (1 kb extended both sides). (E) Venn diagrams representing OsCLSY4-dependent 21-22 nt sRNAs. (F) Boxplots representing abundance of 21-22 nt sRNAs from OsCLSY4 dependent 21-22 nt sRNA loci (lost and gained, respectively). *-significant. ns-non-significant (Wilcoxon test $p < 0.01$). (G) Venn diagram showing overlap between OsCLSY4 dependent 21-22 nt and 23-24 nt sRNA loci (1 kb extended on both sides). (H) Venn diagrams representing OsCLSY3-dependent 21-22 nt sRNAs. (I) Boxplots representing abundance of 21-22 nt sRNAs from OsCLSY3 dependent 21-22 nt sRNA loci (lost and gained, respectively). (J) and (K) Venn diagrams representing overlap between OsCLSY4 dependent 21-22 nt, 23-24 nt sRNA loci (1 kb extended both sides) and overlap between OsCLSY3 and OsCLSY4 dependent 21-22 nt sRNA loci, respectively (1 kb extended on both sides).
