# Supplementary material for: Loss of function of chromatin remodeler OsCLSY4 leads to RdDM-mediated mis-expression of endosperm-specific genes affecting grain qualities
Source: PLoS Genet. 2025 Dec 1;21(12):e1011956. doi: 10.1371/journal.pgen.1011956 (PMC12680349; doi:10.1371/journal.pgen.1011956)
Supplement: S4 Fig — (PDF) [file pgen.1011956.s004.pdf]

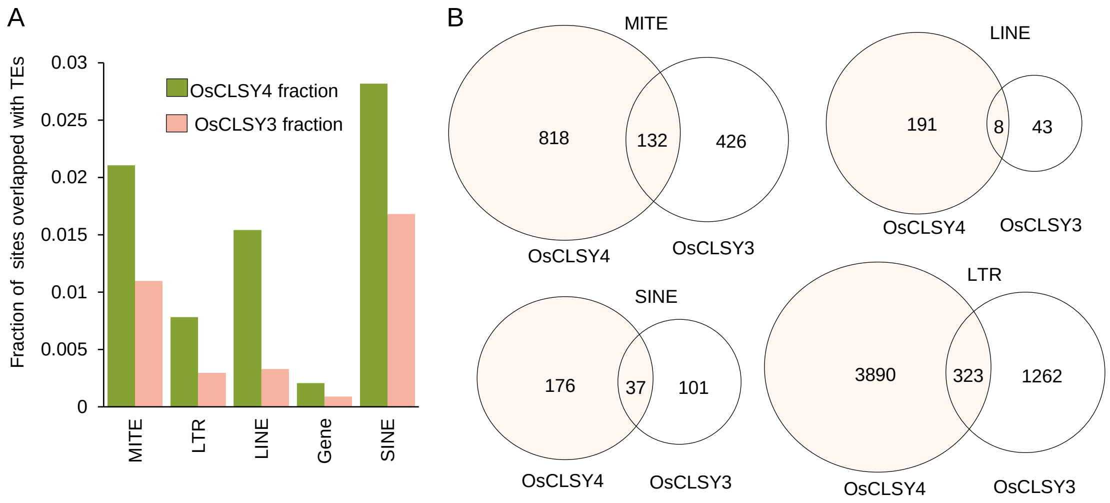

S4 _Fig: Genomic distribution and overlap of OsCLSY3- and OsCLSY4-dependent sRNA loci with different genomic features.

(A) Bar plot showing fraction of OsCLSY4-dependent and OsCLSY3-dependent sRNAs overlapping specific TE features. (B) Venn diagrams showing the overlap of TEs associated with OsCLSY4- and OsCLSY3-dependent sRNA loci.
