# Supplementary material for: Loss of function of chromatin remodeler OsCLSY4 leads to RdDM-mediated mis-expression of endosperm-specific genes affecting grain qualities
Source: PLoS Genet. 2025 Dec 1;21(12):e1011956. doi: 10.1371/journal.pgen.1011956 (PMC12680349; doi:10.1371/journal.pgen.1011956)
Supplement: S5 Fig — (PDF) [file pgen.1011956.s005.pdf]

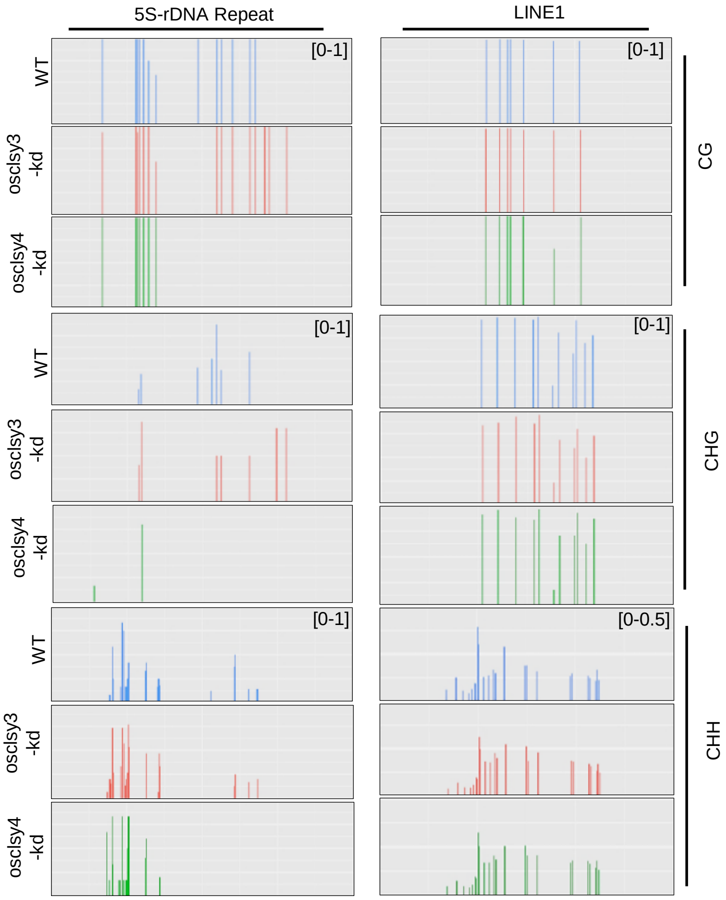

S5 _Fig: DNA methylation in an RdDM loci regulated by OsCLSY4 in leaf.
Targeted bisulfite PCR showing DNA methylation of 5S rDNA repeats and LINE1 TEs in osclsy3-kd and osclsy4-kd leaves.
