# Supplementary material for: Loss of function of chromatin remodeler OsCLSY4 leads to RdDM-mediated mis-expression of endosperm-specific genes affecting grain qualities
Source: PLoS Genet. 2025 Dec 1;21(12):e1011956. doi: 10.1371/journal.pgen.1011956 (PMC12680349; doi:10.1371/journal.pgen.1011956)
Supplement: S6 Fig — (PDF) [file pgen.1011956.s006.pdf]

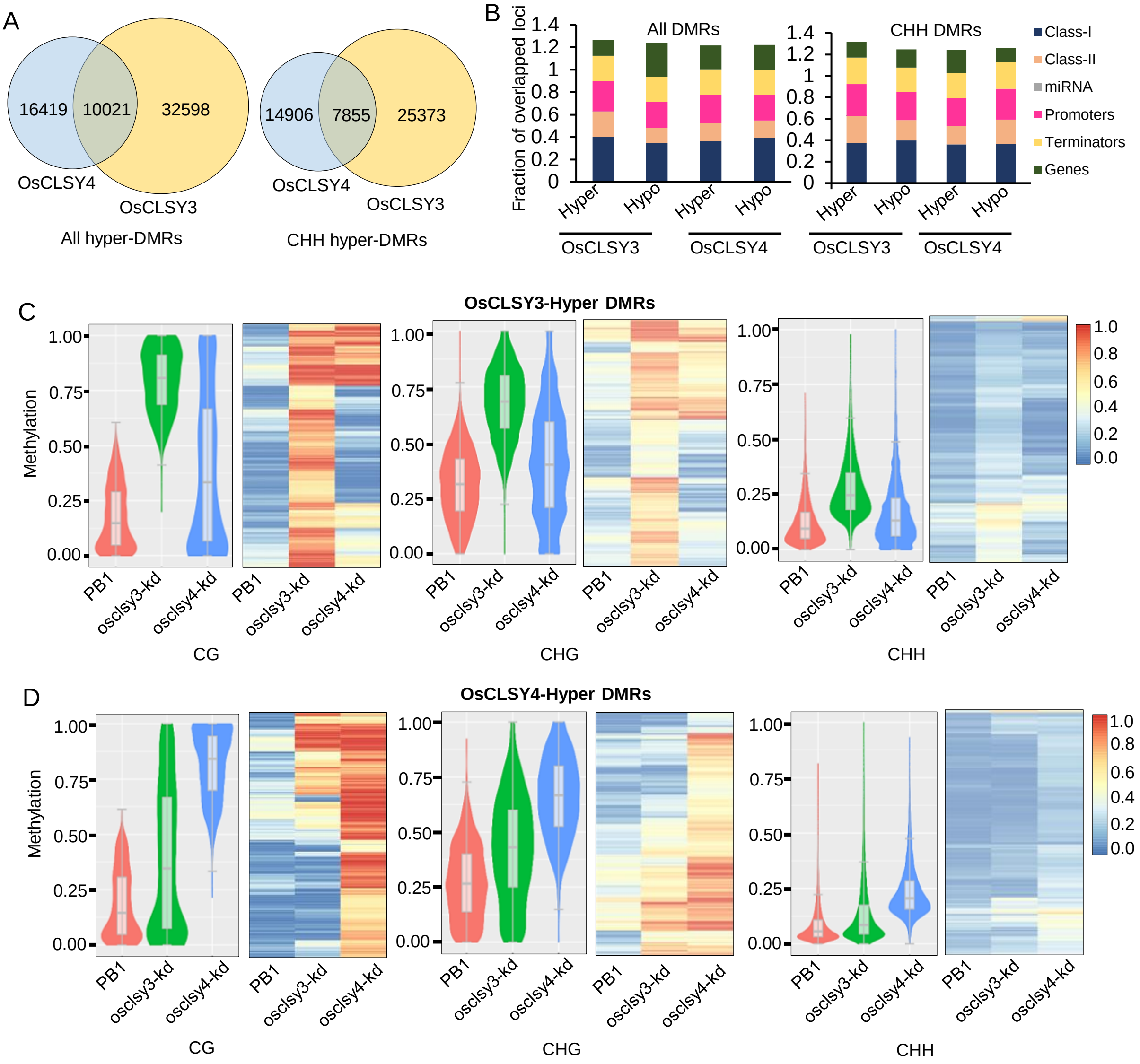

S6 _Fig: OsCLSYs regulate DNA methylation in endosperm non-redundantly. (A) Venn diagrams representing overlap between OsCLSY4 and OsCLSY3 hyper-DMRs. (B) Plots depicting genomic features of OsCLSYs hypo- and hyper-DMRs. (C), (D) Violin-plots and heatmaps showing OsCLSY3 and OsCLSY4-dependent hyper-DMRs, respectively.
