# Supplementary material for: Loss of function of chromatin remodeler OsCLSY4 leads to RdDM-mediated mis-expression of endosperm-specific genes affecting grain qualities
Source: PLoS Genet. 2025 Dec 1;21(12):e1011956. doi: 10.1371/journal.pgen.1011956 (PMC12680349; doi:10.1371/journal.pgen.1011956)
Supplement: S7 Fig — (PDF) [file pgen.1011956.s007.pdf]

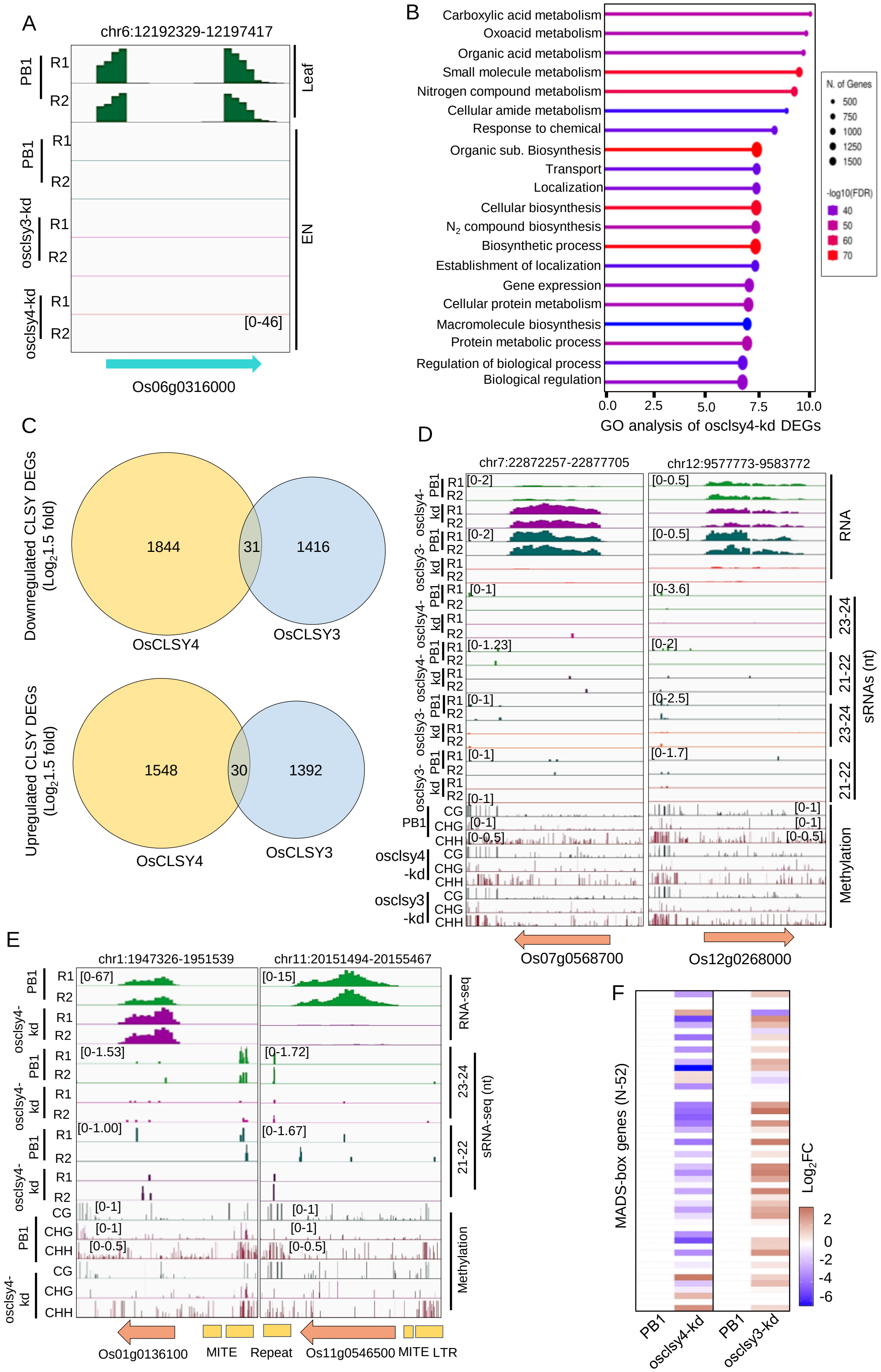

S7 _Fig: OsCLSY4 regulates expression of protein coding genes. (A) IGV screenshots showing lack of expression of green tissue-specific gene in EN transcriptomes. (B) GO analysis of osclsy4-kd EN. (C) Venn diagrams showing overlap between downregulated and upregulated DEGs in genotypes. (D) IGV screenshots showing expression of two DEGs in osclsy3-kd and osclsy4-kd EN. (E) IGV screenshots showing expression of an upregulated and downregulated genes in osclsy4-kd EN. (F) Heatmap showing expression of MADS-box genes in osclsy4-kd and osclsy3-kd EN.
