# Supplementary material for: Loss of function of chromatin remodeler OsCLSY4 leads to RdDM-mediated mis-expression of endosperm-specific genes affecting grain qualities
Source: PLoS Genet. 2025 Dec 1;21(12):e1011956. doi: 10.1371/journal.pgen.1011956 (PMC12680349; doi:10.1371/journal.pgen.1011956)
Supplement: S8 Fig — (PDF) [file pgen.1011956.s008.pdf]

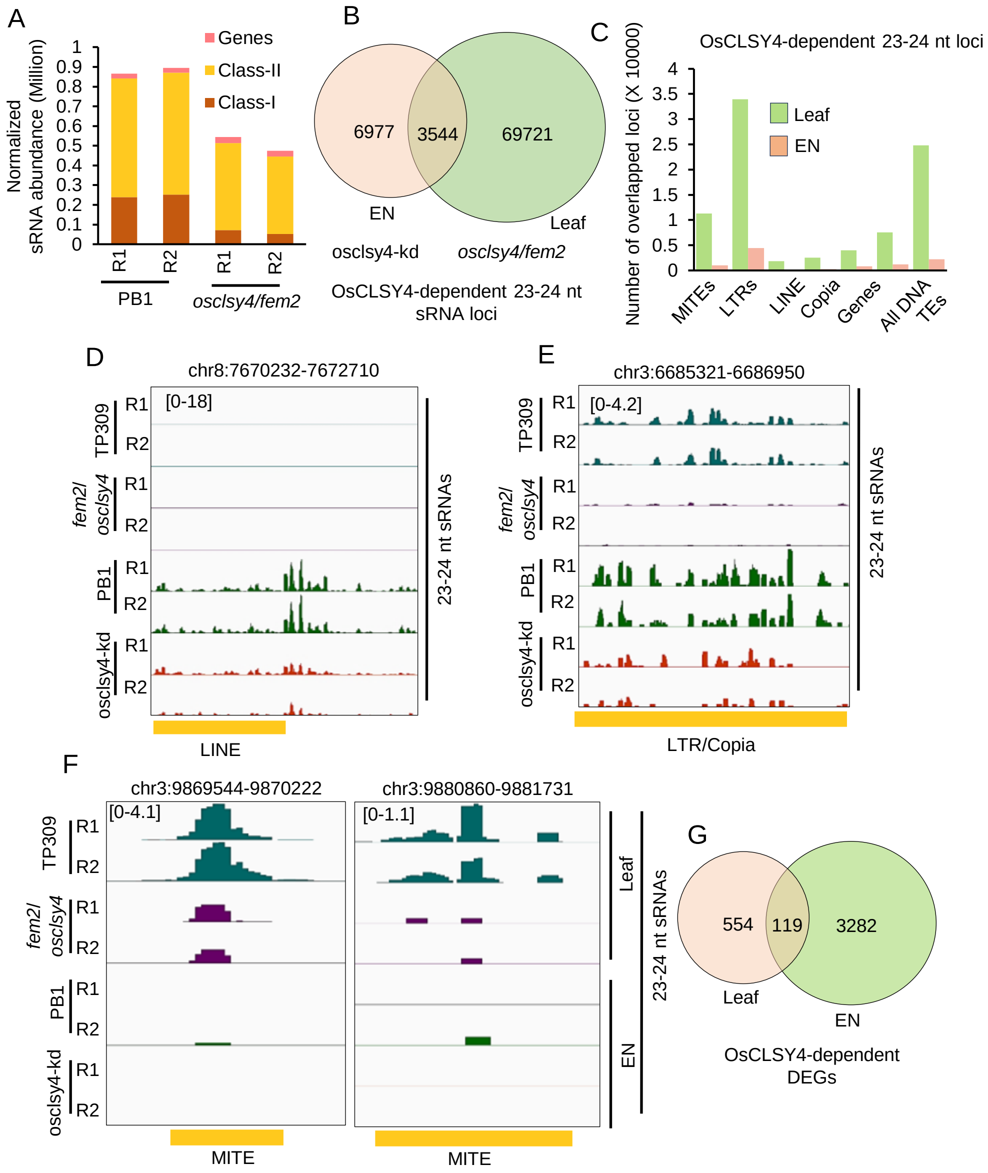

S8 _Fig: OsCLSY4 targets different regions for sRNA production in seedling tissue. (A) Plot showing 23-24 nt sRNA abundance across different genomic features in *osclsy4*/*fem2* seedling tissue. (B) Venn diagrams showing overlap between OsCLSY4-dependent 23-24 nt sRNA loci in EN and leaf. (C) Bar plot representing the overlap of OsCLSY4-dependent 23-24 nt sRNA loci with distinct genomic regions. (D), (E), (F) IGV screenshots showing 23-24 nt sRNA expression in TEs and repeats in leaf and EN in *fem2* and osclsy4-kd lines. (G) Venn diagram showing overlap between *fem2* and osclsy4-kd DEGs.
