# Supplementary material for: Loss of function of chromatin remodeler OsCLSY4 leads to RdDM-mediated mis-expression of endosperm-specific genes affecting grain qualities
Source: PLoS Genet. 2025 Dec 1;21(12):e1011956. doi: 10.1371/journal.pgen.1011956 (PMC12680349; doi:10.1371/journal.pgen.1011956)
Supplement: S1 Table — (DOCX) [file pgen.1011956.s009.docx]

S1_Table: Details of high-throughput genomics data generated in this study

| Sl. No | Dataset type | Genotype | Replicate | Source tissue | GSM Number | GSE Number | Total number of mapped reads obtained | Sequencing mode |
| --- | --- | --- | --- | --- | --- | --- | --- | --- |
| 1 | Small RNA-seq | WT (PB1) | Rep1 | 20 days  Endosperm | GSM8790179 | GSE289382 | 19374449 | Paired end |
| 2 | Small RNA-seq | WT (PB1) | Rep2 | 20 days  Endosperm | GSM8790180 | GSE289382 | 16241812 | Paired end |
| 3 | Small RNA-seq | osclsy4-kd | Rep1 | 20 days  Endosperm | GSM8790181 | GSE289382 | 21233703 | Paired end |
| 4 | Small RNA-seq | osclsy4-kd | Rep2 | 20 days  Endosperm | GSM8790182 | GSE289382 | 19169541 | Paired end |
| 5 | RNA-seq | WT (PB1) | Rep1 | 20 days  Endosperm | GSM8785346 | GSE289150 | 26610703 | Paired end |
| 6 | RNA-seq | WT (PB1) | Rep2 | 20 days  Endosperm | GSM8785347 | GSE289150 | 26447919 | Paired end |
| 7 | RNA-seq | osclsy4-kd | Rep1 | 20 days  Endosperm | GSM8785348 | GSE289150 | 24060165 | Paired end |
| 8 | RNA-seq | osclsy4-kd | Rep2 | 20 days  Endosperm | GSM8785349 | GSE289150 | 27848043 | Paired end |
| 9 | RNA-seq | OsCLSY3OE | Rep1 | 20 days  Endosperm | GSM8785350 | GSE289150 | 12797491 | Paired end |
| 10 | RNA-seq | OsCLSY3OE | Rep2 | 20 days  Endosperm | GSM8785351 | GSE289150 | 11505785 | Paired end |
| 11 | Targeted bisulfite | WT Leaf (PB1) | Rep1 | 60 days Leaf | GSM8785386 | GSE289154 | 528089 | Paired end |
|  | Targeted bisulfite | WT Leaf (PB1) | Rep2 | 60 days Leaf | GSM8785387 | GSE289154 | 570046 | Paired end |
| 12 | Targeted bisulfite | osclsy4-kd Leaf | Rep1 | 60 days Leaf | GSM8785388 | GSE289154 | 698626 | Paired end |
|  | Targeted bisulfite | osclsy4-kd Leaf | Rep2 | 60 days Leaf | GSM8785389 | GSE289154 | 549683 | Paired end |
| 13 | Targeted bisulfite | osclsy3-kd Leaf | Rep1 | 60 days Leaf | GSM8785390 | GSE289154 | 567914 | Paired end |
|  | Targeted bisulfite | osclsy3-kd Leaf | Rep2 | 60 days Leaf | GSM8785391 | GSE289154 | 513113 | Paired end |
| 14 | Bisulfite-Seq | osclsy4-kd Endosperm | NA | 20 days Endosperm | GSM8785379 | GSE289152 | 87951323 | Paired end |
