# Supplementary material for: Loss of function of chromatin remodeler OsCLSY4 leads to RdDM-mediated mis-expression of endosperm-specific genes affecting grain qualities
Source: PLoS Genet. 2025 Dec 1;21(12):e1011956. doi: 10.1371/journal.pgen.1011956 (PMC12680349; doi:10.1371/journal.pgen.1011956)
Supplement: S2 Table — (DOCX) [file pgen.1011956.s010.docx]

S2_Table: Details of high-throughput genomics data obtained from publicly available datasets

| Sl.No | Dataset type | Genotype | Source tissue | SRA number | GSE number | Reference |  |  |
| --- | --- | --- | --- | --- | --- | --- | --- | --- |
| 1 | RNAseq | WT (PB1) | pre-emerged panicle | SRX11493038 | GSE180457 | [1] |  |  |
| 2 | RNAseq | WT (PB1) | pre-emerged panicle | SRX11493039 | GSE180457 | [1] |  |  |
| 3 | RNAseq | WT (PB1) | Anther | SRX11493042 | GSE180457 | [1] |  |  |
| 4 | RNAseq | WT (PB1) | Anther | SRX11493043 | GSE180457 | [1] |  |  |
| 5 | RNAseq | WT_T1_r1 | Leaf | SRX6976682 | GSE138705 | [2] |  |  |
| 6 | RNAseq | WT_T1_r2 | Leaf | SRX6976683 | GSE138705 | [2] |  |  |
| 7 | RNAseq | Nip_se_rep1 | Seedling | SRX5724238 | GSE130168 | [3] |  |  |
| 8 | RNAseq | Nip_se_rep2 | Seedling | SRX5724239 | GSE130168 | [3] |  |  |
| 9 | RNAseq | WT_base_rep1 | Shoot base of seedling | SRX5846194 | GSE131319 | [4] |  |  |
| 10 | RNAseq | WT_base_rep2 | Shoot base of seedling | SRX5846195 | GSE131319 | [4] |  |  |
| 11 | RNAseq | WT_base_rep3 | Shoot base of seedling | SRX5846196 | GSE131319 | [4] |  |  |
| 12 | RNA-seq | Embryo_Rep1 | 25 days Embryo | SRX20001598 | GSE229959 | [5] |  |  |
| 13 | RNA-seq | Embryo_Rep2 | 25 days Embryo | SRX20001599 | GSE229959 | [5] |  |  |
| 14 | RNA-seq | Mature_endosperm_Rep1 | 25 days Endosperm | SRX20001600 | GSE229959 | [5] |  |  |
| 15 | RNA-seq | Mature_endeosperm_Rep2 | 25 days Endosperm | SRX20001601 | GSE229959 | [5] |  |  |
| 16 | RNA-seq | Young_endosperm_Rep1 | 15 days Endosperm | SRX20001602 | GSE229959 | [5] |  |  |
| 17 | RNA-seq | Young_endosperm_Rep2 | 15 days Endosperm | SRX20001603 | GSE229959 | [5] |  |  |
| 18 | RNA-seq | WT_CLSY3_Endosperm_Rep1 | 20 days Endosperm | SRX20001606 | GSE229959 | [5] |  |  |
| 19 | RNA-seq | WT_CLSY3_Endosperm_Rep2 | 20 days Endosperm | SRX20001607 | GSE229959 | [5] |  |  |
| 20 | RNA-seq | KD_CLSY3_Endosperm_Rep1 | 20 days Endosperm | SRX20001604 | GSE229959 | [5] |  |  |
| 21 | RNA-seq | KD_CLSY3_Endosperm_Rep2 | 20 days Endosperm | SRX20001605 | GSE229959 | [5] |  |  |
| 22 | RNA-seq | Nip, rep3 | 18 days old seedling | SRX17907626 | GSE215853 | [6] |  |  |
| 23 | RNA-seq | Nip, rep4 | 18 days old seedling | SRX17907627 | GSE215853 | [6] |  |  |
| 24 | RNA-seq | fem2-3, rep1 | 18 days old seedling | SRX17907628 | GSE215853 | [6] |  |  |
| 25 | RNA-seq | fem2-3, rep2 | 18 days old seedling | SRX17907629 | GSE215853 | [6] |  |  |
| 26 | RNA-seq | fem2-3, rep3 | 18 days old seedling | SRX17907630 | GSE215853 | [6] |  |  |
| 27 | Small RNA-seq | WT_CLSY3_Endosperm_Rep1 | 20 days  Endosperm | SRX20001558 | GSE229958 | [5] |  |  |
| 28 | Small RNA-seq | WT_CLSY3_Endosperm_Rep2 | 20 days  Endosperm | SRX20001559 | GSE229958 | [5] |  |  |
| 29 | Small RNA-seq | KD_CLSY3_Endosperm_Rep1 | 20 days  Endosperm | SRX20001556 | GSE229958 | [5] |  |  |
| 30 | Small RNA-seq | KD_CLSY3_Endosperm_Rep2 | 20 days  Endosperm | SRX20001557 | GSE229958 | [5] |  |  |
| 31 | Small RNA-seq | TP309_seedling_R1 | 18 days old seedling | SRX11502899 | GSE130166 | [3] |  |  |
| 32 | Small RNA-seq | TP309_seedling_R2 | 18 days old seedling | SRX11502900 | GSE130166 | [3] |  |  |
| 33 | Small RNA-seq | fem2-1 | 18 days old seedling | SRX17907624 | GSE215854 | [3] |  |  |
| 34 | Small RNA-seq | fem2-3 | 18 days old seedling | SRX17907625 | GSE215854 | [3] |  |  |
| 35 | Bisulfite-Seq | WT_Endosperm | 20 days Endosperm | SRX23802523 | GSE260651 | [5] |  | Paired end |
| 36 | Bisulfite-Seq | clsy3-kd_Endosperm | 20 days Endosperm | SRX23802524 | GSE260651 | [5] |  |  |
| 37 | Bisulfite-Seq | TP309_seedling_BS | 18 days old seedling | SRX9211924 | GSE158710 | [7] |  |  |
| 38 | Bisulfite-Seq | GAS_seedling_BS | 18 days old seedling | SRX9211925 | GSE158710 | [7] |  |  |
| 39 | Bisulfite-Seq | fem2-1_BS-seq | 18 days old seedling | SRX17907644 | GSE215855 | [3] |  |  |
| 40 | Bisulfite-Seq | fem2-3_BS-seq | 18 days old seedling | SRX17907645 | GSE215855 | [3] |  |  |

**Reference**

1. Hari Sundar G V, Swetha C, Basu D, Pachamuthu K, Raju S, Chakraborty T, et al. Plant polymerase IV sensitizes chromatin through histone modifications to preclude spread of silencing into protein-coding domains. Genome Res. 2023. doi:10.1101/gr.277353.122

2. Hu D, Yu Y, Wang C, Long Y, Liu Y, Feng L, et al. Erratum for: Multiplex CRISPR-Cas9 editing of DNA methyltransferases in rice uncovers a class of non-CG methylation specific for GC-rich regions. Plant Cell. 2022;34: 1416.

3. Wang L, Zheng K, Zeng L, Xu D, Zhu T, Yin Y, et al. Reinforcement of CHH methylation through RNA-directed DNA methylation ensures sexual reproduction in rice. Plant Physiol. 2022;188: 1189–1209.

4. Xu L, Yuan K, Yuan M, Meng X, Chen M, Wu J, et al. Regulation of rice tillering by RNA-directed DNA methylation at miniature inverted-repeat transposable elements. Mol Plant. 2020;13: 851–863.

5. Pal AK, Gandhivel VH-S, Nambiar AB, Shivaprasad PV. Upstream regulator of genomic imprinting in rice endosperm is a small RNA-associated chromatin remodeler. Nat Commun. 2024;15: 7807.

6. Xu D, Zeng L, Wang L, Yang D-L. Rice requires a chromatin remodeler for Polymerase IV-small interfering RNA production and genomic immunity. Plant Physiol. 2023. doi:10.1093/plphys/kiad624

7. Zheng K, Wang L, Zeng L, Xu D, Guo Z, Gao X, et al. The effect of RNA polymerase V on 24-nt siRNA accumulation depends on DNA methylation contexts and histone modifications in rice. Proc Natl Acad Sci U S A. 2021;118: e2100709118.
