# Supplementary material for: Loss of function of chromatin remodeler OsCLSY4 leads to RdDM-mediated mis-expression of endosperm-specific genes affecting grain qualities
Source: PLoS Genet. 2025 Dec 1;21(12):e1011956. doi: 10.1371/journal.pgen.1011956 (PMC12680349; doi:10.1371/journal.pgen.1011956)
Supplement: S3 Table — (DOCX) [file pgen.1011956.s011.docx]

S3_Table: List of oligos and probes used in this study

| Oligo Name | Oligo ID | Oligo sequence (5’-3’) | Purpose | Reference |
| --- | --- | --- | --- | --- |
| STN_OsRAC1_F | 2563 | GCTATGTACGTCGCCATCCAGG | RT-qPCR | [1] |
| STN_OsRAC1_R | 2564 | TGAGATCACGCCCAGCAAGG | RT-qPCR | [1] |
| STN_OsGAPDH_qPCR_F | 3873 | GGGTATTCTGGGTTACGTTGAGGAG | RT-qPCR | [1] |
| STN_OsGAPDH_qPCR_R | 3874 | ACGGATCAGGTCAACAACGCGAGAG | RT-qPCR | [1] |
| AP_CLSY4_RT_F | 3419 | gtggtggatccagtgaatgaagagttgg | RT-qPCR | [1] |
| AP_CLSY4_RT_R | 3420 | cccaaaattatcaccgtcgtcatcagc | RT-qPCR | [1] |
| miRNA168 | 32 | gtcgccgagaagatcctccatc | sRNA northern | [2] |
| U6_probes | 13 and 14 | ggccatgctaatcttctctgtatcgtt and ccaattttatcggatgtccccgaagggac | sRNA northern | [2] |
| MITE siRNA | 3430 | ggtcccacctgtcatacacacact | sRNA northern | [2] |
| AP_CLSY4_amiR1_probe | 3880 | GAGAGGTCATTCGTGCTTACA | sRNA northern | [1] |

References

1. Pal AK, Gandhivel VH-S, Nambiar AB, Shivaprasad PV. Upstream regulator of genomic imprinting in rice endosperm is a small RNA-associated chromatin remodeler. Nat Commun. 2024;15: 7807.

2. Hari Sundar G V, Swetha C, Basu D, Pachamuthu K, Raju S, Chakraborty T, et al. Plant polymerase IV sensitizes chromatin through histone modifications to preclude spread of silencing into protein-coding domains. Genome Res. 2023. doi:10.1101/gr.277353.122
