# Supplementary material for: Loss of function of chromatin remodeler OsCLSY4 leads to RdDM-mediated mis-expression of endosperm-specific genes affecting grain qualities
Source: PLoS Genet. 2025 Dec 1;21(12):e1011956. doi: 10.1371/journal.pgen.1011956 (PMC12680349; doi:10.1371/journal.pgen.1011956)
Supplement: S4 Table — (DOCX) [file pgen.1011956.s012.docx]

S4_Table: Table for methylation non-conversion rate

| Chloroplast | Samples | Non-conversation rate | Bisulfite conversion efficiency (1-rate) |
| --- | --- | --- | --- |
| 1 | WT | 0.043 | 95.7 % |
| 2 | osclsy3-kd | 0.042 | 95.8 % |
| 3 | Osclsy4-kd | 0.059 | 94.1 % |
| Mitochondria | Samples | Non-conversation rate |  |
| 1 | WT | 0.041 | 95.9 % |
| 2 | osclsy4-kd | 0.041 | 95.9 % |
| 3 | osclsy3-kd | 0.052 | 94.8 % |

Non-conversion rate = (unconverted C reads) / (total C reads at unmethylated sites)

Conversion efficiency = 1- (non-conversion rate)

Ideal conversion rate > 99%
