# Supplementary material for: Loss of function of chromatin remodeler OsCLSY4 leads to RdDM-mediated mis-expression of endosperm-specific genes affecting grain qualities
Source: PLoS Genet. 2025 Dec 1;21(12):e1011956. doi: 10.1371/journal.pgen.1011956 (PMC12680349; doi:10.1371/journal.pgen.1011956)
Supplement: Attachment — Submitted filename: Reply_to_reviewers_plosgenetics.docx [file pgen.1011956.s023.docx]

**Reply to Reviewer’s comments**

**Reviewer #1:** This paper follows previous work (from the authors and others) outlining the role of OsCLSY proteins in leaves and the role of OsCLSY3 specifically in endosperm. This manuscript focuses on the role of OsCLSY4 in endosperm and how its function overlaps or not with OsCLSY3. The authors observe that there are both unique and shared sites impacted by these related proteins. Unfortunately, they are unable to directly assess redundancy without a double knockdown mutant. The paper includes analysis of sRNA accumulation (sRNAseq), DNA methylation (whole genome bisulphite sequencing), and transcriptomes (RNAseq). The analyses are routine and include some limited integration of datatypes. The primary conclusion is that although OsCLSY3 is specifically expressed in endosperm it is not the sole CLSY protein functioning in this tissue. This finding will interest researchers focused on the mechanism of RdDM, but does not offer deep insights into the process.

**Suggestions for improvement:**

The manuscript is a bit sloppy and could use careful proof-reading on top of editing for clarity and concision. It rambles quite a bit in the introduction and discussion and it is sometimes difficult to find the important conclusions in the results section. The discussion section is highly speculative in places. For example, “Amino acid variations between both OsCLSY3 and OsCLSY4 between different rice accessions as well their expression variations might differentially regulate TEs and repeats thereby controlling key agronomically important traits” is not based on any particular evidence presented in the paper. The discussion is also quite long and includes a lot of extra detail (eg, explaining the chalky phenotype of endosperm).

**Author’s reply:** Thank you for your insightful suggestions, encouraging comments and elaborate summary of our work. We appreciate your observations and have carefully addressed each and every question and incorporated all the suggestions.

**Reviewer's question:** Please check reference numbering. For example, at the top of **pg 5**, I believe this should be **Ref 32, not 31.**

**Author’s reply:** Thank you for pointing out this error. The correct reference is ref 32 and we have made the correction in the revised manuscript.

**Reviewer's question:** The first paragraph of the results is redundant with Pal et al 2024, which includes expression data, protein structure, etc. Novel points (eg, sequence identity comparison to RMR1) are trivial. This would be better if integrated into the introduction.

**Author’s reply:** We agree with your point. We have shortened the first paragraph keeping only the most important details and also incorporated some additional, more relevant information in the introduction section.

**Reviewer's question:** How are the sRNA abundance plots **(Fig 2A, S2A, and S2B)** normalised? It appears that only 5-10% of the sequenced sRNAs are 20-25 nt in length? In **Fig S2**, panels A and B are also inconsistent. Panel A shows <0.05 as the abundance of 24-nt sRNAs, but panel B shows 0.25. Similarly, 21- and 22-nt sRNAs are 0.01-0.15 in panel A, but approx 0.07 in panel B.

**Author’s reply:** Thank you for this query. Please note that the data in Fig. 2A, S2A and S2B are not percentage plots. We counted aligned reads for each 21, 22, 23 and 24-nts sRNAs separately and then normalized each one using the total number of aligned reads from 16-35 nt. The Y axis shows the normalized reads counts (in millions) for each size group. In the revised version, we changed sRNA abundance (Million) into **Normalized sRNA abundance (Million)**. For further clarification, we attached a table below (Table 1) with details of aligned sRNA reads in each size class.

**Table 1: Numbers of aligned reads (21/22/23/24/25 nt) in each genotype**

| **Number of nucleotides** | **PB1_R1** | **PB1_R2** | **osclsy4-kd_R1** | **osclsy4-kd_R2** |
| --- | --- | --- | --- | --- |
| 20 | 172927 | 154200 | 156898 | 146581 |
| 21 | 380908 | 308842 | 279318 | 222211 |
| 22 | 367848 | 309047 | 235578 | 209381 |
| 23 | 447414 | 364701 | 240311 | 207592 |
| 24 | 1333340 | 965454 | 407384 | 254166 |
| 25 | 339424 | 276638 | 133212 | 111585 |

**Table 2: Total numbers of aligned 16-35 nt reads in each genotype**

| **Number of nucleotides** | **PB1_R1** | **PB1_R2** | **osclsy4-kd_R1** | **osclsy4-kd_R2** |
| --- | --- | --- | --- | --- |
| 16-35 nt | 30332025 | 25410513 | 30099165 | 26895360 |

**Table 3: Numbers of normalized sRNA reads in each genotype**

| **Number of nucleotides** | **PB1_R1** | **PB1_R2** | **osclsy4-kd_R1** | **osclsy4-kd_R2** |
| --- | --- | --- | --- | --- |
| 20 | 5701.13 | 6068.35 | 5212.70 | 5450.04 |
| 21 | 12557.94 | 12154.10 | 9279.92 | 8262.05 |
| 22 | 12127.38 | 12162.17 | 7826.72 | 7785.02 |
| 23 | 14750.54 | 14352.36 | 7983.97 | 7718.50 |
| 24 | 43958.15 | 37994.27 | 13534.72 | 9450.18 |
| 25 | 11190.28 | 10886.75 | 4425.77 | 4148.85 |

**Reviewer's question:** **Pg 6**: “Among the pools of 21, 22 and 24 nt sRNAs, sRNAs that had 5’ A were specifically reduced in osclsy4-kd” This statement is not supported by the data in **Sup Fig 2B**, which shows strong reduction in 24-nt siRNAs regardless of first nucleotide.

**Author’s reply:** Thank you for this observation and the suggestion. To improve clarity and accuracy, we replaced percentage normalization with absolute read counts for 21, 22 and 24-nt sRNAs. We then calculated how many reads start with each nucleotide (A, U, G, or C) to show the true 5′ nucleotide bias, without the influence of relative scaling. This figure actually depicted both the sRNA reduction as well as 5′ nucleotide bias.

**Reviewer's question:** **Pg 7**: “many of the OsCLSY4-dependent 23-24 nt sRNAs from TEs overlapped with OsCLSY3-dependent loci 23-24 nt loci” is also unsupported by the data shown. **Fig 2G** shows two genome regions and does not quantify overlap between OsCLSY3- and OsCLSY4-dependent loci.

**Author’s reply:** Thank you for pointing this out. We agree that the presentation was unclear. In the revised Fig 2G and in its legend, we now clearly state that both kd lines show sRNA reduction at the highlighted TE loci. We also updated the Results section and the figure legend to clarify this interpretation.

**Reviewer's question:** **Pg 7:** “we analysed published sRNA datasets derived from osclsy3-kd endosperm tissues” Please provide a citation for these data and describe which tissue they represent, so that the reader might immediately understand the comparison you are making.

**Author’s reply:** Thank you for this suggestion. We have included this detail in the revised version.

**Reviewer's question:** **Fig2-3:** What are “ASINH” converted RPKM values?

**Author’s reply:** ASINH scale is similar to a log scale. However, it handles low or zero counts better than log scale. In our datasets, many loci have RPKM values of zero. To avoid arbitrarily adding a constant pseudo count (e.g., log (0+1)), we used ASINH scale which is routinely used for this purpose. We applied ASINH after normalizing the raw reads into RPKM.

**Reviewer's question:** **Fig 3H** is an uncontrolled experiment. How can the authors determine that there is no “preference for a specific feature of TE” when they don’t know what the genome frequency of these elements are? Same concern for **Fig S5B.**

**Author’s reply:** Thank you for this point. In these two figures, we intended to show that the two CLSYs did not show any preference for a particular type of TE. For example, the fraction of OsCLSY3 loci overlapping with all MITEs is similar to that of OsCLSY4 loci. To avoid any confusion, we have rephrased the sentence in the revised manuscript and the figure legend.

**Reviewer's question:** **Sup Fig S4** is superfluous to this story. DNA methylation in leaves of clsy4 mutants is already published and this paper is focused on endosperm.

**Author’s reply:** Thank you for this important comment. We included this data for quality control purposes and are not drawing any conclusions from it. We believe connecting the information with the existing data is relevant and useful.

**Reviewer's question:** There appears to be only a small impact on siren sRNA abundance and almost no effect on DNA methylation in clsy3- or clsy4-kd lines. I would therefore not conclude that “both OsCLSYs play a major role in their regulation.” In Arabidopsis siren sRNAs are almost completely eliminated in clsy3 mutants; there is clearly something different happening in rice, according to this data.

**Author’s reply:** Thank you for pointing this out. The impact on siren loci is not strong because we used kd lines for our analysis. Please note that, in *Arabidopsis,* sRNA levels and DNA methylation were severely impacted in *clsy3,4* double mutant. However, *clsy3* single mutant had more reduction of sRNA and DNA methylation when compared to *clsy4* mutant. Also, in *Arabidopsis*, siren RNAs are not completely eliminated in a single *clsy* mutant. Based on our analysis, we interpret that there is redundancy between two CLSYs at siren loci in rice, as similar to *Arabidopsis*.

**Reviewer's question:** **Pg 15:** “the genes associated with siren loci are key regulators of imprinting, triploid block and post-zygotic reproductive barrier” What is the evidence for this statement?

**Author’s reply:** This statement is supported by previous studies, such as Kirkbride et al., 2019, and Dziasek, K et al., 2024. Those studies found that maternal sRNAs, including siren RNAs, regulate the expression of imprinted genes and endosperm cellularization-related genes such as AGLs. These genes are also mis-expressed during triploid block.

**Reviewer's question:** Data supporting the quality of libraries should be included as a supplement. Number of reads, number mapping to the genome, and (critically!) bisulphite conversions values for each library.

**Author’s reply:** We have included the library size, obtained reads in the Supplemental Table 1. We included these additional details in the revised version text. We believe all these quality control parameters are very important additions to the manuscript.

**Table 1: Details of the sRNA-seq data (16-35 nt)**

| **SL** | **Samples** | **Read obtained after adapter trimming (16-35 nt)** | **Read aligned (16-35 nt)** | **Percentage of aligned reads (%)** |
| --- | --- | --- | --- | --- |
| 1 | WT Rep1 | 6981207 | 6424094 | 92.02 |
| 2 | WT Rep2 | 5707852 | 5256864 | 92.10 |
| 3 | osclsy4-kd_Rep1 | 6911531 | 6110724 | 88.41 |
| 4 | osclsy4-kd_Rep2 | 5999289 | 5319293 | 88.67 |
| 5 | WT Rep1 | 9410892 | 8501770 | 90.34 |
| 6 | WT Rep2 | 7123293 | 6448006 | 90.52 |
| 7 | osclsy3-kd_Rep1 | 10382967 | 9860418 | 94.97 |
| 8 | osclsy3-kd_Rep2 | 6378990 | 6055541 | 94.93 |

**Table 2: Details of the RNA-seq data (After adapter trimming)**

| **SL** | **Samples** | **Read obtained after adapter trimming** | **Read aligned** | **Percentage of aligned reads** |
| --- | --- | --- | --- | --- |
| 1 | WT Rep1 | 25570777 | 25039003 | 97.92 |
| 2 | WT Rep2 | 25411761 | 24883558 | 97.92 |
| 3 | osclsy4-kd_Rep1 | 23115098 | 22519105 | 97.42 |
| 4 | osclsy4-kd_Rep2 | 26772204 | 26086545 | 97.44 |
| 5 | WT Rep1 | 11663222 | 11112435 | 95.28 |
| 6 | WT Rep2 | 13216981 | 12216567 | 92.43 |
| 7 | osclsy3-kd_Rep1 | 12467347 | 12024523 | 96.45 |
| 8 | osclsy3-kd_Rep2 | 11984966 | 11274842 | 94.07 |
| 9 | OsCLSY3OE_Rep1 | 11567543 | 10159303 | 87.83 |
| 10 | OsCLSY3OE_Rep2 | 10571360 | 9284254 | 87.82 |

**Table 3: Methylation conversion rate with respect to chloroplast and mitochondria**

| **Chloroplast** | **Samples** | **Non-conversation rate** | **Conversation rate** |
| --- | --- | --- | --- |
| 1 | WT | 0.043 | 99.957 |
| 2 | osclsy3-kd | 0.042 | 99.958 |
| 3 | Osclsy4-kd | 0.059 | 99.941 |
| **Mitochondria** | **Samples** | **Non-conversation rate** | **Conversation rate** |
| 1 | WT | 0.041 | 99.959 |
| 2 | osclsy4-kd | 0.041 | 99.959 |
| 3 | osclsy3-kd | 0.052 | 99.948 |

=============================================================================

=============================================================================

**Reviewer #2:** In this manuscript, Pal and colleagues studied the role of CLASSY (CLSY) proteins in rice small RNA function, TE silencing and gene regulation. In particular this work seeks to tease apart distinct roles of two related proteins, OsCLSY3 from OsCLSY4. The authors published extensively on the molecular and regulatory functions of OsCLSY3 in Pal et al. 2024 Nat Commun. The current manuscript is focused on a narrower set of analyses using artificial microRNA knockdown in the indica rice line Pusa Basmati-1 (PB1), producing osclsy4-kd lines deficient for OsCLSY4. Endosperm sRNAs are globally reduced in these osclsy4-kd lines compared to PB1. The study includes the standard epigenomics approaches: RNA-seq, sRNA-seq and whole-genome bisulfite sequencing, implicating both OsCLSY3/4 proteins in regulating certain TEs and genes.

As could be expected based on studies in Arabidopsis, loss of OsCLSY4 function affects a subset of 23/24-nt siRNA loci and associated RNA-directed DNA methylation (Zhou et al 2018 Nat Genet.). This follows directly from known functions of CLSY proteins in Pol IV recruitment in flowering plants, including Arabidopsis and rice (Zhou et al 2018; Pal et al. 2024). The specialized function of CLSYs as parental dosage regulators in the rice endosperm has key biological and agricultural implications, which was nicely demonstrated by the authors.

**Author’s reply: We sincerely appreciate your thoughtful feedback, positive remarks and detailed overview of our study. Your observations have been invaluable, and we have responded to your suggestions and questions.**

**Reviewer's question:** I am concerned that recent publications, and the current manuscript, are creating inconsistent names for CLSY proteins in rice. Alternative naming arose in the context of a genetic screen, which referred to FEM2, FEL1 and FEL2 proteins (Xu et al, 2024 Plant Physiol.) rather than simply calling them rice homologs to Arabidopsis CLSYs (Smith et al. 2007). Moreover, the rice CLSYs were already identified and named in detailed phylogenetic work by Trujillo et al. 2018 Mol Biol Evol. Inconsistent names were again used in Pal et al. 2024 (i.e., OsCLSY3(Pal), OsCLSY4 (Pal)). What is called “OsCLSY3” here and in Pal et al. 2024 corresponds to OsCLSY5(Trujillo). By contrast, what is called “OsCLSY4” here and in Pal et al. 2024 was called OsCLSY3(Trujillo) in 2018. The authors should properly cite and discuss this previous phylogenetic work, which first differentiated the Poaceae-specific CLSY5(Trujillo) subfamily from its more broadly conserved CLSY3(Trujillo) paralogs.

Furthermore, the quite specific developmental regulation of OsCLSY3(Pal) in rice--expressed in young endosperm 15 days after pollination--is more consistent with the subfunctionalized role symbolized in the name OsCLSY5(Trujillo). On this account, the broad expression and redundant function of OsCLSY4(Pal), described in the current manuscript, seems more consistent with the original OsCLSY3(Trujillo) name, especially because this protein groups phylogenetically with CLSY3 proteins from species outside of the Poaceae, which all lack the CLSY5 subfamily. Therefore, when the authors state (page 11), “It is important to note that the homologs of rice CLSY3 and CLSY4, i.e., AtCLSY3 and AtCLSY4, also regulate siren loci expression in ovules and endosperm indicating possible conserved functions of this clade of CLSY members,” the distinct OsCLSY3(Pal)=OsCLSY5(Trujillo) subfamily is obscured and misassigned.

Later in the Discussion, the authors state that, “23-24 nt sRNAs were much more reduced in osclsy4-kd than osclsy3-kd which further suggested that ubiquitously expressed OsCLSY4 might be the predominantly involved CLSY in rice, similar to AtCLSY1.” In my view, this simply follows from the result that OsCLSY4(Pal)=CLSY3(Trujillo) has broader expression than the more specialized OsCLSY3(Pal)=OsCLSY5(Trujillo) protein, which evolved more recently and shows more limited expression in the endosperm. Certainly, in 18-d-old seedlings, mutations the disrupt OsCLSY4(Pal)=CLSY3(Trujillo) certainly led to the most dramatic loss of 24-nt siRNAs (Xu et al, 2024 Plant Physiol.). However, I do not think that this makes OsCLSY4(Pal) similar to AtCLSY1 in any deep functional sense, given that AtCLSY1 is not involved in siren siRNA biogenesis in Brassicaceae species.

In summary, I recommend that the authors try to make a better accounting of the evolutionary origins of OsCLSY4(Pal)=CLSY3(Trujillo) and the unfortunate recent shift in nomenclature. Much of the data that the authors present here, and presented previously in Pal et al. 2024, make more sense in light of this particular protein being closely related to Arabidopsis CLSY3 and Maize CLSY3/RMR1 (Hale et al. 2007; Trujillo et al. 2018), rather than the alternative paralogs.

**Author’s reply: Thank you for pointing out this. We completely agree with your concerns. In our study, we developed the idea of the nomenclature about rice CLSYs from the published article “Charophytic Green Algae Encode Ancestral Polymerase IV/Polymerase V Subunits and a CLSY/DRD1 Homolog” (Genome Biology and Evolution. 2024 Jun 4;16(6): evae119)). This one is the recent paper from the same group in 2024 after** Trujillo et al. 2018**. According to their recent publication, the authors divided the CLSYs members into two clades namely CLSY1/CLSY2 and CLSY3/CLSY4. Two rice genes that we used for our study (Pal et al 2024 and this manuscript) belong to CLSY3/4 clade. The rice CLSY gene we identified in Pal et al., 2024 which is more similar to *Arabidopsis* CLSY3 at the amino acid level, we named it as OsCLSY3; whereas the one similar to *Arabidopsis* CLSY4, we named it as OsCLSY4 in our published papers. Added to these are the confusions with names derived from mutant screens, where these CLSY genes are named as FEM/FELs. In light of all these, we have made a number of changes in the revised version, where we have cited all these papers, the names they proposed/used and avoided potential ambiguity with names and the functions of their homologs in other model systems. We believe this is the best we could do, where we have tried to keep the naming as per the established norms, provided information about other suggested names, included details on other functions as a matter of fact.**

**Minor issues and corrections:**

Introduction, paragraph 1: “…the rapid evolution of angiosperms often considered as an abominable mystery”. Consider rephrasing here. In English, “abominable” is reserved for terrifying, unpleasant, or disgusting things. A common example is the “abominable snowman”.

**Author’s reply: Thank you. We rephrased the sentence in our revised manuscript.**

Throughout the manuscript: the definite article “the” is frequently missing. Here are some examples on page 2:

(1) “Unlike gymnosperms, majority of the angiosperm seeds contain a triploid endosperm…” --> “Unlike gymnosperms, _the_ majority of the angiosperm seeds contain a triploid endosperm…”

(2) “Incorporation of paternal genome in the nutrient tissue endosperm…” --> “Incorporation of _the_ paternal genome in the nutrient tissue endosperm…”

(3) “In cereal seeds, starch-filled endosperm is the major food source for humans and

other animals” --> “In cereal seeds, _the_ starch-filled endosperm is the major food source for humans and other animals”

(4) “Multiple studies in model plant Arabidopsis indicated that” --> “Multiple studies in _the_ model plant Arabidopsis indicated that”

**Author’s reply: Thank you for pointing these out. This is very helpful. We have corrected all these sentences in our revised text.**

Introduction, page 2: The authors overuse the verb “regulate” to describe the activities of DNA methyltransferases. METHYLTRANSFERASE1 (MET1) catalyzes 5-methylcytosine maintenance at CG dinucleotides, rather than just “regulating” CG methylation. The same problem arises when the authors state that CMT3, CMT2 or DRM2 “regulate” DNA methylation in particular contexts, because they do not specify what they mean by the verb. “Regulate” suggests the enzymes act in a dynamic process involving differential targeting, developmental timing, and/or responsiveness to particular environmental cues. This is partially true, but it is not really the point of the paragraph. Finally, DRM2 can mediate de novo DNA methylation in all sequence contexts, not just CHH methylation.

**Author’s reply: Thank you for these corrections. We have corrected all of these mistakes in the revised manuscript.**

Results, page 5: “The RdDM pathway plays a crucial role in pollen viability and reproductive success in plants and several mutants in this pathway, such as nrpd1a, clsy3, several AGO genes exhibit pollen sterility [21,32,40].” In Arabidopsis thaliana and Capsella rubella species, this “nrpd1a” mutation should be referred to as “nrpd1”, because the gene mutation once called “nrpd1b” is now conventionally called “nrpe1”. NRPD1 and NRPE1 are the largest subunits of quite distinct RNA polymerases, Pol IV and Pol V, not redundant a / b paralogs forming the same enzyme. Also, “several AGO genes” is at odds with the logical flow in this sentence. The authors were referring to mutants, not genes, at the outset of their list.

**Author’s reply: Thank you. We have corrected all of these errors in our revised manuscript.**

We thank both the Reviewers for the comments and suggestions. We believe these comments and suggestions have immensely helped us to improve the manuscript.
