# Supplementary material for: Loss of function of chromatin remodeler OsCLSY4 leads to RdDM-mediated mis-expression of endosperm-specific genes affecting grain qualities
Source: PLoS Genet. 2025 Dec 1;21(12):e1011956. doi: 10.1371/journal.pgen.1011956 (PMC12680349; doi:10.1371/journal.pgen.1011956)
Supplement: Attachment — Submitted filename: Reply_to_Reviewer_Plosgen2.docx [file pgen.1011956.s024.docx]

Reviewer #1: I am disappointed that the authors’ have made few changes in response to the reviewers’ suggestions. In particular, there continue to be many problems in the manuscript, including issues that were raised in the first round of review.

**Reviewer's question:** The authors cannot state CLSY3 and CLSY4 “do not show any preference for a particular type of TE” without comparison to genome averages in Fig 3H. They can conclude that CLSY3 and CLSY4 have *the same* preferences for genomic features, but they have not defined these preferences. For example, if 80% of the OsCLSY3 targets were LINE elements and 80% of the OsCLSY4 targets were also LINE elements, it would be obvious that both proteins have a preference for LINEs, because the genome is not 80% LINE element. But I cannot judge whether 5-10% MITEs and ~30-40% LTRs is enrichment or depletion of these elements relative to the genome. I can only conclude that there is no difference between CLSY3 and CLSY4 preference.

**Author’s reply:** We thank the Reviewer for this insightful comment. We have now quantified enrichment analysis of Transposable elements (TEs) by comparing base pair (bp) overlaps of OsCLSY3 and OsCLSY4 targeted loci with annotated TEs relative to randomly shuffled loci of equal size and chromosomal distribution.

Our results show that OsCLSY4 loci are significantly enriched for SINEs LINEs MITEs and LTRs, while OsCLSY3 loci are enriched for SINE and MITEs. These results demonstrate that both proteins targets TEs but differ in the spectrum of TEs they preferentially regulate.

To access TE enrichment, we performed the following analysis:

1. We computed the total number of base pairs occupied by each TE class (MITE, LINE, LTR) in the rice reference genome to establish the genome-wide distribution.
2. We calculated the number of base pairs (bp) in the genome overlapped by CLSY4- and CLSY3-dependent loci with each TE class, and also for shuffled loci.
3. Calculated enrichment (in bp) overlapped by CLSY loci/ total bp of that TE class in genome. Same calculation was also performed for shuffled loci.
4. To test significance, we compared the observed overlaps to obtained shuffled loci using Fisher’s extract test (p<0.01).

This analysis helped us to identify preferentially targeted regions by OsCLSY4 loci relative to genome wide expectations and helped us to compare them with CLSY3-dependent loci. We also overserved that the sets of TEs overlapped by OsCLSY4 are largely different from those overlapped by OsCLSY3.

The corresponding figure has been added in the revised manuscript (please see the figure attached below), and text has been updated to reflect these observations.

B

A

C

**Fig.**

(A) Bar plots showing fraction of CLSY4-dependent and CLSY3-dependent sRNAs overlapping specific TE features, compared to the fraction obtained from an equal number of shuffled loci across the same TEs. Overlapping regions are measured in base pairs (bp). In plots, *-significant (Fisher’s exact test, p < 0.01). (B) Bar plot showing fraction of CLSY4-dependent and CLSY3-dependent sRNAs overlapping specific TE features. (C**)** Venn diagrams showing the overlap of TEs associated with CLSY4 and CLSY3-dependent sRNA loci**.**

**Reviewer's question:** I do not know how the authors conclude that “among the pools of 21, 22, and 24 nt sRNAs, sRNAs that had a 5’ A were specifically reduced in osclsy4-kd” (Sup Fig 2B). It is clear from the revised figure that sRNAs with a 5’A (brown bars) are slightly reduced at the 21-nt size, reduced by about half in the 22-nt size, and reduced by at least 75% in the 24-nt class. Clearly sRNAs with a 5’ A were not *specifically* reduced in osclsy4-kd. This is such a minor part of the published story I’m not even sure why it is included, but by misrepresenting their data (even after correction in review), it makes everything look suspect.
**Author’s reply:** We appreciate the Reviewer’s careful assessment. We agree with the point that reduction of 5’A sRNAs are not confined to a single size class but occurs across multiple sRNA size categories, with different degrees of reduction. We have corrected the corresponding statement in the text accordingly.

 In addition, we have plotted the data as a percentage plot to more clearly illustrate the relative changes across size classes (please see the figure attached below).

**Fig.**The plots showing 5’ nucleotide level of 21 to 24 nt sRNAs in PB1 and osclsy4-kd endosperm.

**Reviewer's question:** Similarly, there is no explanation for the inconsistency between Sup Fig 2A and 2B. Both of these plots show “sRNA abundance (Million)” while the response to reviewers says that it is “normalized sRNA abundance (Millions)”. Regardless of the title, I assume the normalization was the same in both plots, yet the scales are quite different. There isn’t even a description of what these are normalized against in the manuscript. As someone in the field, I would be uncomfortable citing this research, since a reader can’t even infer how such analysis was done.
**Author’s reply:** We thank the reviewer for carefully noting this inconsistency. Upon re-examining the data, we found that Supplementary Figures 2A and 2B had been normalized using two slightly different normalization factors in the previous version of the manuscript. The Supplementary Figure 2A was normalized to the total number of aligned 16-35 nt sRNAs, but Supplementary Figure 2B was normalized with total aligned 20-25 nt sRNAs.

Now, we have corrected this error and normalized both datasets using same normalization method, based on total aligned 16-35 nt sRNAs (Normalization factor = 10^6^/ aligned sRNAs 16-35 nt). Supplementary Figure 2A and S2B both are now in same scale. The updated plots are included below (please see the figure attached below).

**Fig.**

(A) Plot showing expression of 21-25 nt sRNAs in PB1 and individual kd lines in EN.

(B) Stacked barplot showing abundance of first 5’ nucleotide of mapped sRNAs in WT and clsy4-kd endosperm.

**Reviewer's question:** Reviewer 2 makes an excellent point about CLSY3(Pal) being a Poaceae-specific duplication (CLSY5 in Trujillo) and CLSY4 resembling the ancestral CLSY3/4. While the revised text is a little more clear about the different naming systems, the idea of a Poaceae-specific duplication is not included. Given the unique endosperm of the grass family and the endosperm-specific expression of CLSY3 (CLSY5), this would seem to be an important point.
**Author’s reply:** Thank you for this valuable suggestion. We have included both of the points in the revised manuscript. Specifically, we have incorporated a statement about the *Poaceae*-specific duplication of CLSY3/4 clade.

To avoid the nomenclature issue, we added the locus IDs (MSU and RAPDB) in all three *CLSY* genes in introduction. Please see the highlighted text in revised version.

**Reviewer's question:** Thank you for the information about “ASINH” normalization. I assume ASINH is “Hyperbolic arcsine scaling”. I recommend that you define this somewhere in the manuscript, since it is not clear to this reader and probably many PLoS Genetics readers.
**Author’s reply:** We have included this information in the method section. Please see the highlighted text.

**Reviewer's question:** The bisulfite conversion data (and other HTS data information) is essential and should be provided in the manuscript, not just to the reviewers. I also am puzzled by the addition of a conversion rate and a non-conversion rate. I have only seen this applied in mammalian systems where the conversion rate analyses only CG dinucleotides (which are not expected to convert in mammalian cells), while non-conversion rate applies to non-CG sites. If that were applied to the chloroplast and mitochondrial sequences, then these samples have 4-5% non-conversion (bisulfite failure) and are therefore acceptable, but borderline. This is important information for readers and should be included, but the authors must only report the meaningful rate - the rate of non-converted cytosines.

**Author’s reply:** Thank you for this insightful suggestion. We agree that bisulfite conversion data are essential and we have now included in the revised Supplemental Tables (Please see the Supplementary Table 4 in revised Supplemental information). For our samples, the non-conversation rates (4-6%) correspond to 94-96% conversion efficiency, which is acceptable though slightly below optimal levels (ideal >99%). This clarification and data presentation have been added to the revised supplemental Table 4 and methods section.

Reviewer #2:

**Reviewer's question:** In this revision, Pal and colleagues significantly improved their manuscript, which focuses on functional differences between the CLASSY (CLSY) proteins they call CLSY3 and CLSY4 in rice. The authors addressed most of the reviewers’ main concerns. The nomenclature problem, in which inconsistent names were assigned to proteins variously called “CLSY3”, “CLSY4” or “CLSY5” in past studies, remains quite difficult to resolve in the context of the current study. The authors have explained the rationale for their alternative choices. I do recommend that they re-read the manuscript, however, to correct some remaining English language issues.
**Author’s reply:** Thank you for the supportive comments and your encouragement. We have carefully re-read and edited the text. We included the gene IDs of CLSYs in the revised text.

Minor corrections:

Abstract: “RNA-directed DNA methylation (RdDM) sequence-specifically targets transposable elements (TEs)…” I recommend that they rephrase this sentence to, “In plants, RNA-directed DNA methylation (RdDM) targets transposable elements (TEs) and repeats in a sequence-specific manner, often including tissue-specific factors” …or similar for better readability.

Pg3, paragraph 2: “Although, clsy1,2 and clsy3,4 double mutants regulated comparatively more number of sRNA loci than single mutants, suggesting some degree of redundancy within the CLSY family proteins [26,27,35]”, should start, “However, clsy1,2 and clsy3,4 double mutants affected a greater number of sRNA loci…”

Pg4, paragraph 1: “a clear mechanistic understanding has been eluded,” should be “a clear mechanistic understanding has been elusive.”

Pg5, paragraph 1: “N-terminal intrinsically-disorder regions, …” needs correcting to “N-terminal intrinsically disordered regions, …”

Pg6, paragraph 2: “class I (TEs that replicate in genome by copy paste mechanism) … class II (… cut paste mechanism)”, is typically written as follows, “class I (TEs that replicate in the genome by a copy-and-paste mechanism) … class II (… cut-and-paste mechanism).”

Pg18, paragraph 1: “We did not observe any preference of either CLSYs for specific features”. Grammatical correction: “We did not observe any preference of either CLSY for specific features.”

Generally, it is helpful to include line numbers in reviewer copies to facilitate corrections.

**Author’s reply:** Thank you for pointing out those errors. We have corrected those in the revised text. The line numbers have been included.

We sincerely thank both the Reviewers for their valuable comments and suggestions. These have helped greatly to reduce the inconsistencies and improved the readability of the manuscript.
